# Recurrent Deliberate Foreign Body Ingestion: Psychiatric Comorbidities, Management Strategies, and Ethical Considerations

**DOI:** 10.1007/s11920-026-01712-1

**Published:** 2026-08-14

**Authors:** Christina V. Raghunandan, Elizabeth Hoffman, Heather Schultz

**Affiliations:** Department of Psychiatry, University of Michigan Medical School, University of Michigan, 1500 E. Medical Center Dr, Ann Arbor, Michigan 48109 USA

**Keywords:** Deliberate foreign body ingestion, Borderline personality disorder, Self-injurious behavior, Consultation-liaison psychiatry, Dialectical behavior therapy, Medical ethics

## Abstract

**Purpose of Review:**

Recurrent deliberate foreign body ingestion (DFBI) is a high-risk, resource-intensive behavior most often associated with underlying psychiatric conditions. Although medical and surgical complications are well documented, the psychiatric drivers and optimal management of DFBI are less well characterized. This review synthesizes current evidence on the epidemiology, psychiatric comorbidities, behavioral mechanisms, management strategies, and ethical challenges associated with DFBI [1, 2, 4, 6].

**Recent Findings:**

Recent work highlights the roles of emotion dysregulation, personality pathology—particularly borderline personality disorder—and reinforcement mechanisms in perpetuating recurrent ingestion [1, 3, 10]. A small subset of individuals accounts for a disproportionate share of hospitalizations, underscoring the chronic and relapsing nature of this behavior and its substantial healthcare costs [1, 4]. Effective care requires coordinated multidisciplinary management that integrates medical stabilization with psychiatric treatment, including structured behavioral plans, consistent team-based responses, and attention to countertransference [2, 7]. Psychotherapeutic modalities, particularly dialectical behavior therapy and borderline personality disorder–informed models, are central to long-term management, while pharmacologic interventions primarily target comorbid conditions rather than ingestion [10–12]. Ethical considerations, including assessment of decision-making capacity, perceptions of futility, and resource allocation, further complicate care [13–15].

**Summary:**

DFBI is a complex, recurrent behavior driven by diverse psychiatric, behavioral, and systemic factors. Effective management requires coordinated multidisciplinary care that integrates medical stabilization, psychiatric treatment, structured behavioral interventions, and ethical decision-making to reduce recurrence and improve outcomes.

## Introduction

Deliberate foreign body ingestion (DFBI) refers to the intentional ingestion of non-nutritive objects and represents a complex clinical phenomenon at the intersection of psychiatry, medicine, and ethics [1, 2]. While some episodes occur in the context of suicidal intent, the majority are non-suicidal and instead reflect underlying psychiatric pathology and maladaptive coping mechanisms, often conceptualized as forms of non-suicidal self-injury (NSSI) [1, 3]. Patients with DFBI frequently present to emergency departments, often repeatedly, resulting in significant healthcare utilization, procedural interventions, and financial burden [1, 4].

Historically, published work on DFBI has been dominated by gastroenterology and surgical perspectives, reflecting the immediate medical risks and need for endoscopic or surgical intervention [2, 4]. However, emerging evidence underscores the importance of psychiatric formulation in understanding and managing recurrent ingestion [2, 3, 7]. This review aims to provide a comprehensive, psychiatry-focused synthesis of DFBI, emphasizing recent findings in comorbidities, behavioral mechanisms, treatment strategies, and ethical challenges.

### Case Illustration

An adult patient with a history of mood and eating disorders, psychological trauma, and personality pathology presented with deliberate foreign body ingestion (DFBI) complicated by serious gastrointestinal injury requiring repeated medical and procedural intervention. Self-injurious behavior initially involved ingestion of medication with suicidal intent during periods of acute distress but, over time, progressed to recurrent ingestion of foreign objects—including household items and small hardware—resulting in frequent emergency presentations and multiple endoscopic interventions, consistent with patterns described in high-utilizing DFBI cohorts [1, 4].

Episodes were typically preceded by marked affective dysregulation and interpersonal conflict, followed by transient psychological relief and increased engagement with medical staff, aligning with reinforcement models of self-injury and DFBI [3, 7]. The patient reported compulsive urges and intrusive thoughts to ingest non-food items, as well as deriving validation and comfort from the patient role. Although the patient believed these symptoms reflected obsessive-compulsive disorder, longitudinal assessment suggested that personality pathology characterized by emotional instability, unstable relationships, identity disturbance, and impulsive self-harm provided a more parsimonious formulation.

Following medical stabilization, the patient required ongoing medical and psychiatric hospitalization. Despite continuous observation, environmental restriction of potential ingestible objects, and a structured behavioral care plan, DFBI episodes persisted, necessitating repeated collaboration with gastroenterology and surgery and multiple endoscopic retrievals, consistent with reports of chronic, relapsing DFBI even in highly structured settings [3, 6].

The psychiatric team adopted a consistent, non-punitive approach emphasizing dialectical behavior therapy (DBT)–informed strategies, including distress tolerance, emotion regulation, and interpersonal effectiveness skills. Daily goal setting, collaborative medication adjustments targeting mood and impulsivity, and efforts to enhance the patient’s sense of autonomy were associated with partial reductions in the frequency and medical severity of episodes, though chronic risk remained. Discharge planning required close coordination with outpatient providers and recognition that, outside of a controlled environment, the patient would likely remain at high risk for recurrence, mirroring longitudinal descriptions of DFBI as a persistent, resource-intensive behavior driven by complex psychiatric and systemic factors [3, 7].

### Epidemiology and Clinical Burden

DFBI is an under-recognized but recurrent form of self-injury, most frequently seen in patients with severe mental illness, personality disorders, or intellectual disability, and in institutional settings such as prisons and forensic hospitals [1–3]. Across settings, a small number of individuals account for a disproportionate share of episodes and admissions. In Kaazan et al.’s multicentre Australian cohort, 35 mental health patients generated 166 DFBI-related admissions between 2013 and 2020, with more than half exhibiting repetitive presentations and a mean of 3.3 foreign bodies per episode [1]. Tromans et al. identified 27 inpatients within forensic mental health and intellectual disability services responsible for 133 incidents over one year, approximately one event every 2.7 days, with a single patient contributing 24 incidents [2]. Earlier series similarly demonstrate clustering among a psychiatrically complex subgroup, many with high rates of personality pathology [2–4].

This pattern results in frequent emergency presentations, endoscopic procedures, and readmissions, placing a substantial burden on healthcare systems [1, 4]. DFBI is associated with a range of complications, including mucosal injury, impaction, obstruction, perforation, hemorrhage, and need for surgical intervention, particularly with high-risk ingestions or delayed presentation [2–4]. However, length of stay is often driven less by procedural complexity and more by observation needs, ongoing self-harm risk, and challenges in discharge planning [1, 4]. Collectively, these findings highlight DFBI as a chronic, relapsing condition shaped by psychiatric, behavioral, and systems-level factors rather than an isolated gastroenterological problem [1–4].

### Medical Complications and Financial Costs

DFBI carries meaningful risks of gastrointestinal and systemic complications while also generating substantial healthcare expenditures. In the retrospective series by Huang et al., 33 individuals accounted for 305 admissions over eight years, with total costs exceeding US $2 million, largely driven by hospital bed-days rather than procedural complications; the average length of stay was 5.6 days, and endoscopy itself was rarely complicated [4]. Kaazan et al. similarly reported 680 cumulative inpatient days among a small cohort of patients, with endoscopy frequently required and an increasing trend in DFBI-related admissions over time [1].

Studies in prison and forensic settings further demonstrate that DFBI is often repetitive and resource-intensive, requiring recurrent imaging, endoscopy, and occasional surgery despite involving relatively few individuals [2–4]. Systematic reviews confirm that DFBI frequently necessitates repeated medical interventions and is associated with substantial financial burden, although cost-effectiveness data for preventive strategies remain limited [2–4].

Clinically, most ingestions can be managed endoscopically or conservatively; however, high-risk ingestions and delayed presentation increase the likelihood of serious complications, including obstruction, perforation, fistula formation, hemorrhage, and sepsis [2–5]. While severe outcomes occur in a minority of cases, recurrent ingestion in patients with complex psychiatric comorbidities has been associated with prolonged hospitalizations, repeated invasive interventions, and ethically challenging decisions regarding the proportionality of care [5, 9–11].

These findings reinforce prior epidemiologic observations of concentrated burden within a high-risk subgroup, which drives a disproportionate share of both complications and costs, underscoring the importance of integrated psychiatric, behavioral, and systems-level interventions to reduce recurrence and healthcare burden [1–4, 9].

### Psychiatric Comorbidities: Recent Findings

Several overlapping clinical subtypes have been proposed, including a widely cited four-category framework encompassing pica, psychotic disorders, malingering, and personality pathology [2, 3, 11]. Pica-related ingestion, more commonly recognized in children but also seen in adults, may be associated with nutritional deficiencies, intellectual disability, autism spectrum disorder, or obsessive–compulsive spectrum phenomena [1, 11]. In psychotic disorders, ingestion may occur in response to delusional beliefs or command hallucinations, with limited appreciation of medical risk [2, 4, 11]. Malingering-driven DFBI is typically observed in highly structured settings (e.g., prisons, forensic or inpatient units), where ingestion functions to obtain secondary gain such as transfer, respite from an aversive environment, or other instrumental outcomes [2, 4]. Finally, in patients with personality disorders, particularly borderline and antisocial traits, DFBI often represents a form of severe behavioral dysregulation and self-injury, analogous to cutting or scratching, occurring in the context of affective crises, perceived abandonment, or interpersonal conflict [2, 4, 11].

Across these groups, DFBI may serve multiple psychological functions: modulation of unbearable affect or dissociative states, enactment of self-punishment, communication of distress, or pursuit of care, validation, and the “sick role” through the need for urgent medical or surgical intervention [2, 4, 11]. Some reports also describe experiences of relief or even euphoria linked to sedation or anesthesia during endoscopic retrieval, potentially reinforcing the behavior [1, 11]. Clinically, patients frequently have extensive histories of psychiatric comorbidity, prior self-harm, and high healthcare utilization, underscoring the need to identify the primary drivers of ingestion and to formulate DFBI within each patient’s broader developmental, diagnostic, and relational context [2, 4, 11].

### Mechanisms and Behavioral Drivers

Recent conceptual models suggest that DFBI often functions as a form of maladaptive coping, analogous in many respects to NSSI [3, 8, 9]. In individuals with BPD and related conditions, ingestion may serve to regulate affect, reduce psychological distress, or transform internal emotional pain into a concrete, somatic experience [3, 8–10]. Some patients report dissociative states or a sense of automatism around ingestion episodes, like descriptions in the broader self-harm literature [9, 10].

Operant reinforcement mechanisms—including increased attention, intensive monitoring, perceived protection, and relief following medical intervention—may further perpetuate the behavior, particularly when health-care responses are inconsistent or highly reactive [7, 8]. In institutional settings, secondary gain (e.g., changes in unit placement, increased privileges, or temporary removal from aversive environments) may also contribute, although this is rarely the sole or primary driver [6, 8].

Additionally, emerging evidence and theoretical work suggest that repetitive ingestion behaviors may be maintained through habit formation, altered reward processing, and neurobiological vulnerabilities similar to those implicated in other compulsive or self-injurious behaviors, though empirical data remain limited [9–11]. Understanding these mechanisms is critical, as it shifts the focus from purely behavioral containment to addressing underlying psychological drivers and system responses.

## Management Strategies: Current Approaches

### General Principles and Systems of Care

Management of DFBI presents significant clinical and systems-level challenges. Healthcare teams frequently experience overwhelm, uncertainty, compassion fatigue, and frustration, underscoring the importance of team support and consistent care approaches [1–4].

Management is best approached through coordinated, multidisciplinary care. Recent literature emphasizes the need for standardized protocols across emergency departments, medical units, and psychiatric settings to reduce care variability, avoid unnecessary endoscopy or surgery, and minimize inadvertent reinforcement of maladaptive behaviors [14, 15]. Core components include early identification of high-risk patients, structured environmental restriction of ingestible objects, and implementation of appropriate observation levels (e.g., 1:1 monitoring) when clinically indicated [6–8]. Collaboration among psychiatry, gastroenterology, surgery, emergency medicine, nursing, and security personnel is essential to ensure consistent messaging, limit-setting, and responses to ingestion or threats of ingestion [1–4, 6–8].

Psychiatric consultation-liaison teams play a central role in framing DFBI within a trauma- and personality-informed model, addressing countertransference, supporting behavioral limit-setting, and educating staff about the chronic, relapsing nature of the behavior and the importance of avoiding secondary gain from prolonged hospitalization. Developing individualized care plans and protocolized pathways that can be shared across encounters and settings has been associated with greater consistency, fewer unnecessary procedures, and more judicious use of inpatient care [1–3].

### Emergency Intervention and Multidisciplinary Approach

Acute management requires prompt risk assessment and coordination across medical and psychiatric teams [4, 6–8]. Protocol-based emergency approaches emphasize early identification of patients with a history of DFBI, rapid imaging when indicated, removal of potential ingestible objects, provision of meals that do not require utensils, and close observation, including 1:1 supervision when warranted [4, 6–8]. Regular interdisciplinary communication and precautionary staff meetings are recommended, given the unpredictable, recurrent nature of ingestion behaviors and the high risk of staff burnout [1–4].

From a medical standpoint, management decisions should be guided by the type, size, and location of the ingested object, as well as the patient’s clinical status [4, 6, 8]. Urgent endoscopic retrieval is indicated for high-risk ingestions such as button or disk batteries lodged in the esophagus, multiple magnets, and sharp metallic objects in the esophagus, stomach, or duodenum [4, 6, 8, 12]. In contrast, conservative management with observation and serial imaging may be appropriate for lower-risk ingestions, even when objects meet “high-risk” criteria established by guidelines, provided careful clinical monitoring and clear plans for escalation are in place [6, 8, 12]. These decisions require individualized risk–benefit assessment to balance procedural risks, radiation exposure, and the potential for serious complications [4, 6, 8].

A comprehensive medical evaluation should also consider micronutrient deficiencies, toxic exposures (e.g., lead), and other contributing medical conditions, particularly in patients with pica or neurodevelopmental disorders [1, 2, 6]. Effective emergency management depends on close collaboration between emergency medicine, gastroenterology, surgery, psychiatry, and nursing teams to ensure timely intervention, consistent communication, and continuity of care across settings, with early involvement of mental health services to address underlying drivers and reduce repeated high-risk presentations [1–4, 6].

Determining the need for inpatient psychiatric hospitalization is a critical early step in the management of patients with DFBI. Although hospitalization may be necessary in certain circumstances, it is not universally indicated and may, in some cases, inadvertently reinforce maladaptive behaviors through increased attention, containment, or access to care. Admission should be strongly considered in the presence of acute safety concerns, including active suicidal ideation with intent or plan, ingestion behavior driven by suicidal intent, severe impulsivity with inability to maintain safety in a less restrictive setting, or comorbid conditions such as acute psychosis, mania, or severe mood destabilization that impair judgment. Conversely, for individuals whose ingestion behaviors are chronic, non-suicidal, and primarily related to personality pathology or reinforcement dynamics, repeated hospitalizations may offer limited therapeutic benefit and should be carefully weighed against potential iatrogenic effects. In such cases, emphasis should be placed on structured outpatient care, consistent behavioral plans, and longitudinal therapeutic engagement.

### Psychotherapeutic Interventions

Among psychotherapeutic modalities, dialectical behavior therapy (DBT) has the strongest evidence base for reducing self-injurious behaviors and suicidal ideation in patients with borderline personality disorder and adolescents with chronic self-harm, targeting emotional dysregulation, distress intolerance, interpersonal difficulties, and impulsive behavior—core domains frequently implicated in DFBI [1–3, 6–8, 10]. Although DFBI-specific data are limited to case reports and small series, DBT-informed approaches have been associated with reductions in ingestion frequency and emergency presentations in patients whose DFBI occurs in the broader context of repetitive self-harm [1–3, 6–8].

Other structured treatments for personality pathology and chronic self-injury, such as Good Psychiatric Management (GPM) and mentalization-based therapy, may also be useful but have not been systematically studied in DFBI [1–3, 6, 10]. Trauma-focused therapies (e.g., trauma-focused CBT, EMDR) may benefit individuals with significant histories of abuse or neglect when delivered after stabilization of acute self-injury and within a framework that prioritizes emotion regulation and safety skills [1–3, 6, 9].

For patients with intellectual disability or autism spectrum disorder, behavioral interventions informed by applied behavior analysis and functional assessment may help identify triggers and modify environmental contingencies that maintain ingestion behavior [2, 6, 8].

Consistent outpatient engagement and continuity of care are critical, as episodic or fragmented treatment is associated with recurrent presentations and greater reliance on acute care. Care pathways that integrate outpatient psychotherapy, crisis planning, and proactive communication with emergency and inpatient teams—ideally via shared care plans or alerts—may help attenuate the cycle of recurrent DFBI and high service utilization [1–4, 6–8, 10].

### Pharmacologic Considerations

Pharmacologic treatment is generally aimed at comorbid psychiatric conditions rather than DFBI itself [1–3, 6, 10]. Selective serotonin reuptake inhibitors (SSRIs) may be helpful for mood and anxiety symptoms and for patients with obsessive–compulsive or ruminative features, while mood stabilizers (e.g., lithium, valproate, lamotrigine) and atypical antipsychotics may reduce impulsivity, affective instability, and psychotic symptoms in selected patients [1–3, 6, 10]. Evidence specific to reducing DFBI frequency is limited to case reports and small series, and pharmacotherapy should therefore be viewed as an adjunct to, not a substitute for, structured psychotherapeutic and behavioral interventions [1–3, 6, 10–12].

Emerging evidence from the broader self-injury and compulsive-behavior literature suggests potential benefit of agents such as naltrexone and N-acetylcysteine (NAC) in reducing urges for repetitive self-harm and related behaviors, although DFBI-specific data remain preliminary [3, 6, 10–12]. NAC has shown promise in small studies of NSSI and compulsive behaviors, possibly through modulation of glutamatergic transmission and reward circuitry, but optimal dosing and long-term efficacy are not yet established [12]. Naltrexone has been used off-label to attenuate the reinforcing properties of self-injury via opioid receptor antagonism, with case-level evidence of benefit but concerns about tolerance and limited data on sustained effects [3, 6, 10–12].

Benzodiazepines may have a role in acute anxiolysis or agitation but carry risks of disinhibition, dependence, and paradoxical worsening of impulsivity and should be used cautiously in individuals with chronic self-harm [1–3, 6, 10]. Across medication classes, treatment should be individualized, regularly reviewed, and embedded within a comprehensive, psychiatry-informed care plan rather than pursued as a stand-alone solution [1–3, 6, 10–12].

## Ethical Considerations

### Autonomy, Capacity, and Coercion: Balancing Autonomy and Protection

DFBI presents complex ethical challenges, particularly in balancing respect for patient autonomy with the duty to prevent serious harm in the context of self-injurious behavior [14, 15]. Autonomy refers to a patient’s right to make informed decisions and to self-govern, while clinicians are simultaneously guided by the principles of beneficence and non-maleficence. In patients with DFBI, repeated ingestion behaviors may lead to significant morbidity, including perforation, need for surgical intervention, and risks associated with repeated anesthesia. As such, clinicians must continuously weigh the risks and benefits of intervention against respect for patient choice.

Central to this process is the assessment of decision-making capacity, commonly guided by the Appelbaum criteria, which evaluate whether a patient can understand relevant information, appreciate the consequences of their decision, reason about treatment options, and communicate a consistent choice [13]. In the context of DFBI, these assessments are particularly nuanced. Patients may appear superficially capable yet demonstrate impaired appreciation of risk due to acute emotional dysregulation, psychosis, or maladaptive coping patterns [13, 15].

In situations where ingestion poses imminent risk of death or catastrophic morbidity—such as ingestion of batteries or sharp objects—patients may lack the capacity to refuse intervention. In such cases, involuntary treatment, including endoscopic or surgical removal, may be ethically and legally justified [13–15]. These decisions should be made on a case-by-case basis, ideally with multidisciplinary input and, when available, ethics consultation [13–15].

DFBI is frequently a manifestation of psychological distress and self-injurious behavior rather than a fully autonomous, rational decision. As behaviors become more repetitive and self-destructive, the ethical tension between respecting autonomy and ensuring safety becomes increasingly pronounced. This raises important questions regarding paternalism and the appropriateness of coercive interventions in clinical care. While clinicians must avoid overly paternalistic approaches, there are circumstances in which protective intervention is warranted to prevent serious harm.

In line with broader medical ethics practice, in chronic and severe cases, particularly when patients demonstrate persistent impairment in insight and judgment, consideration may be given to more structured oversight, including guardianship or other legal mechanisms to support decision-making. Ultimately, ethical management of DFBI requires careful, individual assessment, balancing patient rights with clinician responsibility to protect life and minimize harm.

### Countertransference, Stigma, and Resource Stewardship

Repeated presentations for DFBI can evoke strong emotional responses among healthcare providers, including frustration, helplessness, burnout, and therapeutic pessimism [3, 15]. These countertransference reactions may contribute to inconsistent care, punitive attitudes, minimization of risk, or premature discharge planning. Over time, clinicians may become desensitized to recurrent crises, potentially leading to unconscious bias or underestimation of the seriousness of ingestion events. Patients who frequently present to emergency settings may also be labeled as “manipulative” or “attention-seeking,” reinforcing stigma and negatively impacting care delivery [3, 10, 15].

Conceptualizing DFBI as a manifestation of severe psychological distress and maladaptive coping, rather than willful misconduct, is essential to reframing provider responses and promoting empathic, consistent care [3, 10, 15]. Qualitative and clinical literature emphasizes the importance of staff support, supervision, and education to mitigate countertransference and maintain therapeutic engagement [15]. Institutions should actively address stigma and avoid labeling patients as “untreatable,” particularly given that individuals with mental illness, intellectual disability, and those in custodial settings are disproportionately represented in DFBI populations.

DFBI also raises important questions regarding resource stewardship and equitable allocation of care. A small number of patients often account for a disproportionately high use of medical resources, including repeated hospitalizations, endoscopic procedures, and surgical interventions [4, 15]. This can create ethical tension between responsible resource utilization and the obligation to provide appropriate, evidence-informed care. Ethical frameworks emphasize that all patients deserve equitable treatment regardless of the perceived preventability of their condition or degree of personal responsibility [15].

Clinicians must also grapple with complex questions regarding the appropriateness and limits of repeated intervention. When does the burden of ongoing procedures outweigh potential benefit, and how should care be approached in the context of persistent, treatment-resistant behavior? Despite these challenges, the ethical principle of non-maleficence includes avoiding both overtreatment and the withholding of necessary care. Ensuring that clinical decisions are guided by individualized risk assessment rather than frustration or bias is essential.

Structured, proactive approaches can help balance these competing demands. Development of individualized behavioral care plans, clear treatment agreements, and consistent multidisciplinary responses can reduce variability in care and mitigate escalation during acute presentations [7, 12, 15]. Planning—such as predefined emergency protocols, identification of a consistent treatment team, and pre-emptive staff meetings—can improve coordination and reduce splitting among providers [15]. Emergency clinicians, who are often at the forefront of DFBI management, benefit from clear guidance developed in collaboration with psychiatry and gastroenterology and readily accessible within the medical record.

Ethical analyses emphasize that effective management of DFBI requires a comprehensive assessment of psychosocial drivers, including psychiatric comorbidity, substance use, and interpersonal stressors [14]. Interventions should extend beyond acute medical management to include outpatient psychiatric care, psychotherapy, pharmacologic treatment, and social support systems [14]. Although hospitalization may at times reinforce maladaptive behaviors, it remains necessary in certain cases for acute stabilization and safety monitoring. Ultimately, balancing countertransference, stigma, and resource stewardship requires a structured, compassionate, and ethically grounded approach that prioritizes both patient safety and equitable care (Table 1).

**Table 1:** Ethical principles & clinical applications in the management of recurrent foreign body ingestion

Domain	Clinical Application
Autonomy and Protection from Harm	Assess decision-making capacity using structured frameworks (e.g., Appelbaum criteria), particularly in high-risk ingestions where impaired appreciation or emotional dysregulation may limit autonomy.
Psychological Formulation	Conceptualize ingestion behavior as maladaptive coping rather than willful misconduct to support empathic, consistent, and non-punitive care.
Countertransference	Provide staff support, supervision, and education to mitigate frustration, burnout, and therapeutic pessimism that may lead to inconsistent or biased care.
Stigma and Bias	Refrain from labeling patients as “manipulative” or “attention-seeking,” and emphasize trauma-informed, respectful care, particularly for vulnerable populations.
Resource Stewardship	Deliver appropriate, evidence-informed care regardless of perceived preventability, while balancing resource stewardship with patient-centered decision-making.
Multidisciplinary Care Planning	Implement standardized protocols and individualized behavioral plans with consistent team-based responses to reduce variability and escalation.
Intervention Thresholds	Individualize decisions regarding endoscopic or surgical intervention by balancing procedural risks with the likelihood of serious complications.
Proactive Care Planning	Develop predefined emergency protocols, maintain consistent treatment teams, and ensure clear documentation to improve coordination and reduce splitting.
Longitudinal Psychiatric Care	Address underlying drivers (e.g., psychiatric comorbidity, trauma, substance use) through outpatient treatment, psychotherapy, and social support systems.

## Discussion, Clinical Implications, and Future Directions

DFBI rarely occurs in isolation and is closely linked to underlying psychopathology, most commonly borderline personality disorder and other severe personality disorders, mood disorders, or psychotic spectrum conditions. This behavior is typically repetitive, impulsive, and resistant to intervention, leading to substantial healthcare utilization, medical complications, and ethically challenging decision-making.

This pattern is consistent across epidemiologic studies, with a small subset of individuals accounting for a disproportionate share of hospitalizations and procedures [1–4]. In many cases, ingestion serves a function analogous to NSSI, including regulation of emotional distress, communication of psychological pain, self-punishment, or elicitation of care and validation [2, 3]. Reinforcement mechanisms—such as transient relief of distress and increased staff attention—may further perpetuate this cycle despite patients’ awareness of medical risks.

These observations carry important clinical implications. Clinicians should maintain a high index of suspicion for underlying psychiatric pathology in patients presenting with DFBI, particularly those with recurrent episodes, high-risk ingestions, or complex medical courses. Conceptualizing DFBI as a manifestation of severe psychological distress and impaired emotion regulation supports more compassionate and structured care which may help reduce staff frustration, blame, and therapeutic pessimism.

Early psychiatric involvement and structured care planning are critical, alongside close collaboration across disciplines including psychiatry, gastroenterology, surgery, nursing, and, when applicable, security staff. Clear communication, consistent boundary-setting, and ongoing staff education are essential to maintaining team cohesion and mitigating burnout. DBT-informed approaches remain central to treatment, targeting affective dysregulation, shame, and interpersonal instability that frequently underlie DFBI [10]. Pharmacologic interventions may help address comorbid mood symptoms, impulsivity, and psychotic features but appear to have limited direct impact on ingestion behavior itself and should be embedded within a broader psychosocial treatment plan.

Ethical considerations further complicate management. Recurrent ingestion challenges the balance between respecting autonomy and ensuring patient safety, particularly when behavior carries risk of life-threatening complications such as perforation, hemorrhage, or sepsis. Decision-making capacity assessments, often guided by the Appelbaum criteria, are essential in this population [13]. In some cases, patients may meet criteria for involuntary treatment to prevent serious harm, raising questions about appropriate limits of coercion and the proportionality of repeated invasive interventions. Countertransference, stigma, and perceptions of futility may contribute to inconsistent care, premature discharge, or avoidance of necessary intervention, underscoring the importance of institutional support, ethics consultation, and protocol-driven approaches.

Future work should prioritize the development and evaluation of standardized, integrated care models that bridge emergency, medical, psychiatric, and custodial settings. There is a critical need for controlled studies examining the effectiveness of psychotherapeutic interventions such as DBT and related approaches in reducing DFBI frequency, healthcare utilization, and patient-centered outcomes. Further investigation into psychological and neurobiological mechanisms—including reward processing, habit formation, and trauma-related factors—may inform more targeted interventions. Additionally, research addressing ethical and system-level challenges, including capacity assessment, thresholds for coercive intervention, staff support, and resource stewardship, will be essential to guide clinical practice and policy for this high-risk population.

## Conclusion

Recurrent DFBI is a complex, high-risk behavior that demands a multidisciplinary and psychiatry-informed approach. Rather than viewing DFBI solely as a behavioral or medical problem, framing it as a manifestation of emotional dysregulation, maladaptive coping, and underlying psychopathology allows for more effective and compassionate care. Integrating psychiatric, medical, and ethical perspectives—alongside structured care plans and consistent team-based approaches—can help reduce recurrence, improve clinical outcomes, and support healthcare providers managing this challenging population. Continued research and development of integrated care models will be essential to advancing treatment and addressing the substantial individual and system-level burden associated with DFBI.

## Key References

- Kaazan P, Seow W, Tan Z, et al. Deliberate foreign body ingestion in patients with underlying mental illness: a retrospective multicentre study. *Australas Psychiatry*. 2023;31:619–624.
  - ⚬ This multicenter study is important because it provides contemporary epidemiologic data on recurrent DFBI and demonstrates the substantial healthcare utilization associated with a small group of high-frequency presenters.
- Alli-Balogun O, Singh G, Korenis P. Intentional foreign body ingestion in a patient with borderline personality disorder and other comorbidities. *Prim Care Companion CNS Disord*. 2023;25:22cr03287.
  - ⚬ This case report highlights the relationship between borderline personality disorder, emotional dysregulation, and recurrent ingestion behavior, emphasizing the importance of psychiatric formulation and multidisciplinary management.
- Vourliotis C, Ng F, Bruxner G. “Fighting for the last gasp”—severe borderline personality disorder and posttraumatic stress disorder, chronic deliberate ingestion of foreign bodies, and palliative struggles in intensive care. *J Psychiatr Pract*. 2023;29:150–156.
  - ⚬ This article is notable for its description of a particularly severe and medically complex case of recurrent DFBI and provides valuable insight into the challenges of managing treatment-resistant ingestion behaviors.
- Briner R, Frossard JL. Recurrent intentional foreign body ingestion in psychiatric patients: an ethical issue. *Forensic Sci Add Res*. 2024;6.
  - ⚬ This recent article provides a focused discussion of the ethical issues surrounding recurrent DFBI, including decision-making capacity, autonomy, coercive interventions, and resource stewardship.

## Data Availability

No datasets were generated or analysed during the current study.
